# Social media use and disordered eating in young adults: the protective and risk pathways of physical activity and body image

**DOI:** 10.3389/fpsyg.2025.1699756

**Published:** 2026-01-14

**Authors:** Wei Yu, Liwen Cui, Changli Liu, Jian Wang, Jianye Li, Mariusz Lipowski

**Affiliations:** 1 School of Sport Education, Tianjin University of Sport, Tianjin, China; 2 Faculty of Sports and Arts, Harbin Sport University, Harbin, China; 3 Department of Physical Education, Heilongjiang University of Science and Technology, Harbin, China; 4 College of Physical Education, Qinghai Minzu University, Xining, China; 5 Faculty of Physical Education, Gdansk University of Physical Education and Sport, Gdańsk, Poland; 6 Department of Physical Education, Shanxi Medical University, Jinzhong, China; 7 Faculty of Social and Humanities, WSB Merito University in Gdansk, Gdańsk, Poland

**Keywords:** body image dissatisfaction, dietary behavior, parallel mediation model, physical activity, social media use

## Abstract

**Background/objectives:**

Social media is a pervasive force in youth culture, shaping body perception and dietary habits. However, the psychological and behavioral pathways linking social media use to eating behavior remain insufficiently understood. This study examined the mediating roles of body image dissatisfaction and physical activity in the association between social media use and dietary behavior among young adults.

**Methods:**

A cross-sectional survey was conducted among 422 Chinese university students (Mean age = 22.82 ± 2.42) between November 2024 and February 2025. Participants completed validated measures of social media use, body image dissatisfaction (BSQ-16), physical activity (IPAQ-SF), and dietary behavior (DEBQ). Data were analyzed using confirmatory factor analysis (CFA), parallel mediation modeling (PROCESS macro), and ANOVA for subgroup comparisons.

**Results:**

Confirmatory factor analysis confirmed the measurement model (CFI = 0.926, RMSEA = 0.057). Social media use significantly predicted higher body image dissatisfaction (β = 0.578, *p* < 0.001) and lower physical activity (β = −348.717, *p* < 0.001), both of which were associated with disordered eating (β = 0.442 and β = −0.000, respectively). Parallel mediation analysis identified two significant indirect effects: through body image dissatisfaction (indirect effect = 0.256, *p* < 0.001) and physical activity (indirect effect = 0.084, *p* < 0.001). ANOVA showed that lower physical activity was associated with higher social media use and poorer dietary outcomes.

**Conclusion:**

Social media is associated with dietary behavior through dual psychological and behavioral pathways. Interventions should target body image resilience and active lifestyles to mitigate the health risks posed by digital media environments.

## 1 Introduction

In the digital age, social media has become a ubiquitous presence in the daily lives of young people. With platforms such as Instagram, TikTok, Facebook, and Snapchat boasting billions of active users, a growing body of literature has highlighted the pervasive role of social media in shaping psychological states and health-related behaviors (Centola, 2013; Laranjo et al., 2015). Young people, in particular, are both the most avid consumers and the most impressionable targets of social media content, making them especially vulnerable to its psychosocial effects (O’Reilly et al., 2018). Among the various domains influenced by social media, dietary behavior, defined as individuals’ patterns of food choice, meal timing, and nutritional intake, has drawn increasing attention from public health researchers due to its impact on long-term health outcomes such as obesity, eating disorders, and metabolic syndrome (Avenell et al., 2004; Williams et al., 2015).

Evidence suggests that social media platforms facilitate exposure to food-related content, including recipe videos, “mukbang” eating broadcasts, and influencer-endorsed dietary practices (Qin et al., 2025; Hawkins et al., 2021). This exposure often promotes idealized or extreme dietary trends (e.g., detox teas, intermittent fasting, keto diets), which may influence eating behavior through mechanisms of observational learning and social reinforcement (Suwalska and Bogdański, 2021). However, findings are inconsistent: while some studies report associations between social media use and healthier eating intentions (e.g., increased fruit and vegetable consumption), others indicate increased susceptibility to disordered eating patterns, emotional eating, and unhealthy food cravings (May et al., 2012; Rossi, 2025). This inconsistency calls for a more nuanced understanding of the mediating mechanisms linking social media engagement with dietary choices.

One prominent pathway through which social media may influence eating behavior is body image dissatisfaction. Social comparison processes are central in this dynamic. According to Social Comparison Theory (Festinger, 1954), individuals tend to evaluate themselves based on comparisons with others, particularly in the absence of objective standards. On image-centric platforms such as Instagram, curated and often digitally enhanced portrayals of thinness or muscularity become salient referents for comparison (Rousseau, 2024). This can give rise to body dissatisfaction and internalization of unrealistic appearance ideals, particularly among adolescents and young adults (Lawler and Nixon, 2011).

Body image dissatisfaction has consistently been linked to maladaptive eating behaviors such as restrictive dieting, binge eating, and compensatory behaviors (Brytek-Matera and Czepczor, 2017; Pinkasavage et al., 2015). Meta-analyses confirm that exposure to appearance-focused content is associated with both lower body satisfaction and increased drive for thinness or muscularity (Huang et al., 2021). Thus, body image may act as a psychological conduit translating social media exposure into dietary outcomes, an effect that has yet to be fully modeled within a mediation framework incorporating concurrent behavioral pathways.

Simultaneously, social media also shapes physical activity behaviors, which in turn influence dietary regulation and body-related self-evaluation. Exposure to fitness-related content, such as workout routines, transformation stories, and “fitspiration” images, can have dual outcomes. On the one hand, such content may motivate users to adopt healthier routines, increasing exercise frequency and improving dietary control through enhanced self-efficacy and goal setting (Vinnikova et al., 2020). On the other hand, excessive or unrealistic portrayals of ideal bodies may evoke feelings of inadequacy and demotivation, discouraging engagement in physical activity and promoting unhealthy compensatory eating behaviors (Markula, 2001).

The process through which physical activity might mediate the relationship between social media use and dietary behavior warrants further theoretical elaboration. Self-Determination Theory (Deci and Ryan, 2012) emphasizes that individuals are more likely to engage in sustained health behaviors, including exercise and healthy eating, when they feel autonomous, competent, and intrinsically motivated. Social media can either support or undermine these psychological needs, depending on the nature of content and user interpretation (Karahanna et al., 2011; Fox and Moreland, 2015). Thus, exposure to fitness content may contribute to an increased sense of competence and intrinsic motivation, facilitating engagement in physical activity, which may, in turn, promote healthier dietary behavior. Conversely, social media may also expose users to unrealistic ideals that lead to body dissatisfaction, which can disrupt these motivations and potentially promote disordered eating patterns.

Theoretical perspectives such as Objectification Theory (Fredrickson and Roberts, 1997) further elucidate how media-driven exposure to idealized bodies can impact both physical activity and dietary behaviors. Objectification Theory posits that the internalization of objectified body ideals leads to self-surveillance and body monitoring, which can undermine physical and psychological well-being. In the context of social media, exposure to appearance-focused content may contribute to objectification, resulting in heightened body dissatisfaction, diminished self-esteem, and altered health behavior, including physical activity and dietary choices. Therefore, social comparison processes related to both body image and physical activity engagement need to be understood within the broader framework of objectification, as they may simultaneously influence eating and exercise behaviors.

Given these theoretical foundations, this study seeks to investigate the relationship between social media use (independent variable) and dietary behavior (dependent variable) among young individuals, with body image dissatisfaction and physical activity engagement as parallel mediators. This study will utilize a quantitative cross-sectional survey design and structural equation modeling to empirically test the hypothesized mediation paths. By incorporating both psychological and behavioral mediators into a single framework, this study seeks to clarify the dual impact of social media and contribute to the development of targeted digital health interventions. Furthermore, the findings will provide insights for educators, clinicians, and policy-makers seeking to mitigate the adverse effects of media exposure while promoting health-conscious engagement among youth.

**FIGURE 1:**
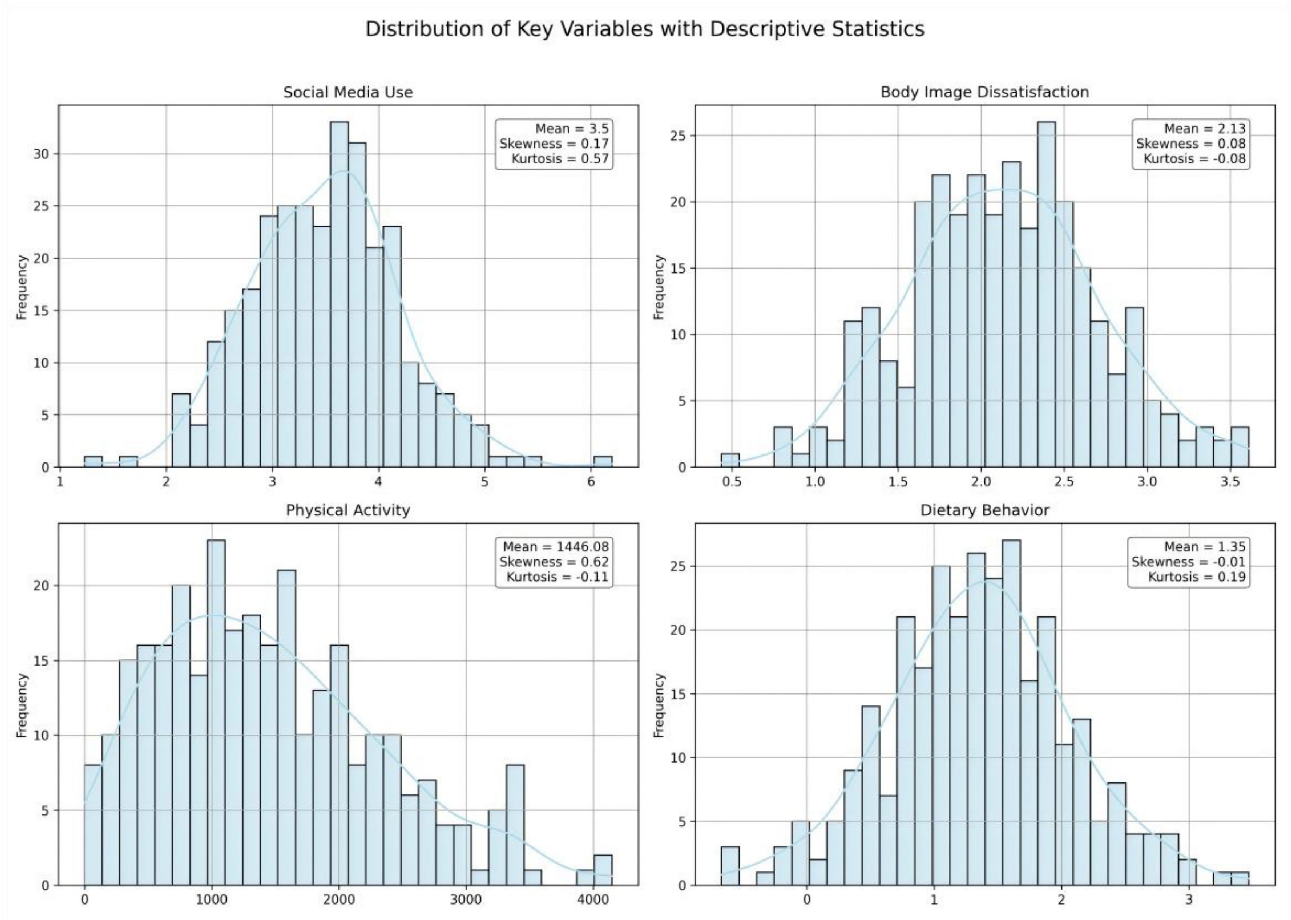
Normal distribution plot.

Building upon the theoretical foundations of Social Comparison Theory, Objectification Theory, and Self-Determination Theory, as well as previous empirical findings, the present study proposes a parallel mediation model to investigate how social media use influences dietary behavior among young individuals through two distinct but simultaneous pathways: body image dissatisfaction (psychological) and physical activity engagement (behavioral). The proposed model is illustrated in Figure 1.

Existing literature has demonstrated a significant association between social media engagement and various aspects of eating behavior, ranging from increased caloric intake and unhealthy snacking to diet restriction and orthorexia, depending on the type of media content and individual interpretation (Chung et al., 2021; Wu et al., 2024). Accordingly, we propose the following direct effect hypothesis:

H1: Social media use is significantly associated with dietary behavior among young people.

Numerous studies indicate that exposure to idealized body images on social media contributes to negative self-perceptions, particularly body dissatisfaction, which in turn influences eating practices such as binge eating, emotional eating, and restrictive dieting (Aubrey, 2006; Merino et al., 2024). This suggests a psychological mediation pathway:

H2a: Social media use is positively associated with body image dissatisfaction.

H2b: Body image dissatisfaction is significantly associated with dietary behavior.

H2c: Body image dissatisfaction mediates the relationship between social media use and dietary behavior.

Simultaneously, physical activity has been shown to influence dietary self-regulation by increasing health consciousness, self-efficacy, and intrinsic motivation for health behavior (Shieh et al., 2015). Social media may both positively and negatively shape users’ engagement with exercise routines depending on their content exposure and interpretation (Goodyear et al., 2018). This gives rise to the behavioral mediation pathway:

H3a: Social media use is significantly associated with physical activity engagement.

H3b: Physical activity engagement is significantly associated with dietary behavior.

H3c: Physical activity engagement mediates the relationship between social media use and dietary behavior.

Given that both body image dissatisfaction and physical activity engagement serve as theoretically distinct mediators, one rooted in internal self-perception and the other in external behavior regulation, it is necessary to test whether they simultaneously mediate the relationship between social media use and dietary behavior. Therefore, the core hypothesis of the present model is:

H4: The relationship between social media use and dietary behavior is simultaneously mediated by (a) body image dissatisfaction and (b) physical activity engagement.

To summarize, the study posits: A direct pathway from social media use to dietary behavior (H1); Two indirect pathways, one via body image dissatisfaction (H2a–H2c) and one via physical activity engagement (H3a–H3c); A parallel mediation model encompassing both mediators (H4). This set of hypotheses will be empirically tested using structural equation modeling (SEM) techniques in a sample of young individuals, with bootstrapping procedures employed to evaluate the significance of indirect effects.

## 2 Materials and methods

### 2.1 Participants

Total of 422 young adults (aged 18–30 years) residing in mainland China were included in this study. All participants were Chinese nationals, recruited from diverse universities across China. The inclusion criteria were as follows:

(1)    aged between 18 and 30 years;
(2)    active engagement with at least one social media platform (e.g., WeChat, QQ, TikTok) at a minimum frequency of once per week in the past 6 months;
(3)    ability to read and complete a standardized questionnaire in either Chinese or English.

Exclusion criteria included:

(1)    a current or previous diagnosis of an eating disorder (e.g., anorexia nervosa, bulimia nervosa);
(2)    any severe psychiatric illness (e.g., schizophrenia, bipolar disorder);
(3)    response bias detected through attention check items, completion time less than 3 minutes, or patterned responses (e.g., straight-lining).

A prior power analysis conducted via G*Power 3.1 indicated that a sample size of at least 200 participants would be required to detect a medium-sized mediation effect (f^2^ = 0.15) with 80% power and α = 0.05 in a model containing two parallel mediators. The final sample size was 445. After eliminating invalid samples, the effective sample size was 422, which exceeded this threshold and ensured that all planned analyses had sufficient statistical power.

### 2.2 Procedure

This study adopted a cross-sectional online survey design. Data were collected over 4 months from November 2024 to February 2025. Recruitment was carried out through university mailing lists, student association portals, and popular social media platforms targeting university student groups and youth communities. The recruitment message contained information about the study objectives, eligibility criteria, and a hyperlink to the online questionnaire.

Participants accessed the survey through a secure Wenjuanxing online platform. Upon entry, they were presented with a detailed informed consent form, which they had to agree to electronically before proceeding. The questionnaire was available in both Chinese and English, allowing respondents to select their preferred language version.

The average time to complete the survey was approximately 10–15 min. The data collection instrument included demographic questions, standardized psychological scales, and embedded attention check items to ensure data quality.

The research protocol was approved by the Harbin Institute of Physical Education, and all procedures adhered to the Declaration of Helsinki and applicable national research ethics guidelines.

### 2.3 Instruments

#### 2.3.1 Social media use

Social media use was assessed using a modified version of the Social Media Engagement Questionnaire (SMEQ) originally developed by Savci and Aysan (2017). The original scale was designed to evaluate the intensity and behavioral engagement with social media platforms. In the present study, the scale was adapted to specifically capture three dimensions relevant to eating-related behaviors: frequency of use, daily duration, and content-specific exposure (e.g., fitness- and food-related posts). The adapted version comprised 8 items, such as “How many hours per day do you spend on social media?” and “How often do you view fitness- or diet-related posts on social media?”. Participants responded using a five-point Likert scale ranging from 1 (Never) to 5 (Very Often). Total scores were obtained by averaging the item scores, with higher values reflecting greater social media involvement and exposure to appearance- and food-related content. This adapted version demonstrated good internal consistency in the present sample, with a Cronbach’s alpha of 0.87.

#### 2.3.2 Body image dissatisfaction

Body image dissatisfaction was assessed using the Body Shape Questionnaire–16 (BSQ-16), a validated short-form version of the original BSQ developed by Evans and Dolan, 1993. The BSQ-16 measures attitudinal concerns and negative evaluations related to body shape and appearance, particularly in the context of perceived weight and shape dissatisfaction. The scale consists of 16 items, including representative statements such as “Have you felt ashamed of your body?” and “Have you avoided situations where you might have to show your body (e.g., swimming or changing clothes)?”. Responses were rated on a six-point Likert scale ranging from 1 (Never) to 6 (Always), with higher total scores indicating greater levels of body image dissatisfaction. The BSQ-16 demonstrated excellent internal consistency in the current sample, with a Cronbach’s alpha of 0.91, indicating high reliability.

#### 2.3.3 Physical activity engagement

Physical activity was assessed using the International Physical Activity Questionnaire – Short Form (IPAQ-SF), a widely validated tool designed to measure self-reported physical activity across multiple domains (Craig et al., 2003). The IPAQ-SF captures frequency (days per week) and duration (minutes per day) of engagement in three types of activity over the previous 7 days: vigorous physical activity, moderate physical activity, and walking. It also includes one item on time spent sitting, although this was not analyzed in the current study.

Following the official IPAQ scoring guidelines, total physical activity was calculated in metabolic equivalent minutes per week (MET-min/week) by multiplying the assigned MET values for each intensity level (vigorous = 8.0 METs, moderate = 4.0 METs, walking = 3.3 METs) by the reported duration and frequency of each activity. These were then summed to produce an overall physical activity score. Higher MET-min/week values reflect greater levels of physical activity engagement. For subgroup comparisons, participants were categorized into three physical activity levels according to standard IPAQ scoring guidelines: Low (<600 MET-min/week), Moderate (600–2,999 MET-min/week), and High (≥3,000 MET-min/week). The IPAQ-SF has demonstrated acceptable reliability and concurrent validity across international populations, including youth and university-aged samples.

#### 2.3.4 Dietary behavior

Dietary behavior was measured using the Dutch Eating Behavior Questionnaire (DEBQ), a widely used instrument developed by Van Strien et al. (1986) to assess eating tendencies associated with emotional regulation, external cues, and dietary restraint. The DEBQ comprises 33 items, divided into three validated subscales: Emotional Eating (13 items), External Eating (10 items), and Restrained Eating (10 items). These subscales reflect distinct behavioral dimensions of eating patterns that may contribute to disordered dietary behavior. Participants rated the frequency of each behavior on a five-point Likert scale, ranging from 1 (Never) to 5 (Very Often). Subscale scores were calculated by averaging relevant items. In the present study, a composite index of disordered dietary behavior was computed by averaging all items across the three subscales, following prior research conventions. Higher composite scores indicate more problematic or dysregulated eating behavior, including tendencies toward emotional overeating, external cue responsiveness, and excessive cognitive control of food intake. The DEBQ demonstrated good internal consistency in this sample, with a Cronbach’s alpha of 0.87.

### 2.4 Data analysis

All statistical analyses were conducted using IBM SPSS Statistics 26.0 and the PROCESS model 6. Preliminary data screening and variable coding were performed in Microsoft Excel prior to analysis.

Descriptive statistics and normality assessment: Descriptive statistics, including means, standard deviations, and skewness/kurtosis, were computed for all continuous variables. Normality was evaluated using both the Kolmogorov–Smirnov test and the Shapiro–Wilk test, alongside visual inspection of histograms and Q–Q plots. Variables that did not meet strict normality assumptions were retained due to the robustness of parametric methods under large samples (*N* = 300).

Reliability analysis: Internal consistency of each questionnaire-based measure (SMEQ, BSQ-16, DEBQ, IPAQ-SF) was assessed using Cronbach’s alpha. All scales demonstrated acceptable to excellent reliability (a > 0.80).

Bivariate correlation analysis: To examine initial associations among key study variables, Pearson’s correlation coefficients were calculated between social media use, body image dissatisfaction, physical activity (MET-min/week), and dietary behavior. Correlation matrices were visualized using a heatmap to aid interpretation.

Parallel mediation analysis: To test the hypothesized parallel mediation model, PROCESS macro Model 4 was employed with social media use as the independent variable (X), dietary behavior as the dependent variable (Y), and body image dissatisfaction and physical activity as parallel mediators (M1 and M2). The analysis estimated: Direct effect (c’) of social media use on dietary behavior; Indirect effects (a_1_b_1_ and a_2_b_2_) via each mediator; Total effect (c) of social media use on dietary behavior. Bootstrapping procedures with 5,000 resamples were used to calculate bias-corrected 95% confidence intervals (CIs) for indirect effects. An indirect effect was considered statistically significant if its CI did not include zero. Effect paths were visualized using a structural mediation diagram, and indirect effect magnitudes were further presented as bar charts.

Group comparisons by physical activity level: Participants were categorized into Low (<600 MET-min/week), Moderate (600–2,999), or High (≥3,000) physical activity groups based on the IPAQ-SF scoring protocol. To compare social media use, body image dissatisfaction, and dietary behavior across physical activity levels, one-way analysis of variance (ANOVA) was performed. Where significant omnibus *F*-tests were detected, *post hoc* comparisons (Tukey HSD or LSD) were conducted. Group differences were visualized via box plots, with significance levels indicated.

## 3 Results

### 3.1 Sample description

Table 1 shows a total of 422 participants were included in the present study. The mean age of the sample was 22.82 years (SD = 2.42), with ages ranging from 18 to 30 years. Regarding gender distribution, 230 participants (56.3%) identified as female and 192 (43.7%) as male. In terms of physical activity level, as assessed using the International Physical Activity Questionnaire (IPAQ) scoring protocol, the majority of participants (*n* = 246, 58.3%) were categorized as having moderate activity levels, while 95 participants (22.5%) fell into the low activity category, and 81 (19.2%) were classified as having high physical activity. These characteristics suggest a relatively balanced and active young adult sample, appropriate for investigating the psychosocial mechanisms linking social media use and dietary behavior.

**TABLE 1:** Sample characteristics (*n* = 422).

Characteristic	Total sample
Age [Mean (SD)]	22.82 (2.42)
**Gender**	
Female	230
Male	192
**Physical activity level**	
High	81
Moderate	246
Low	95

Descriptive statistics and distribution plots were examined for the key variables of the study. As shown in Table 2 and Figure 1, Social Media Use exhibited an approximately normal distribution (Mean = 3.50, Skewness = 0.17, Kurtosis = 0.60). Body Image Dissatisfaction also showed near-normality with minimal skew (Mean = 2.13, Skewness = 0.08, Kurtosis = −0.06). Physical Activity, based on the MET scoring from the IPAQ, displayed a right-skewed distribution (Mean = 1446.08, Skewness = 0.62, Kurtosis = −0.09), which is consistent with prior studies using IPAQ data. Dietary Behavior was presented as normally distributed (Mean = 1.35, Skewness = −0.01, Kurtosis = 0.21).

**TABLE 2:** Descriptive statistics (*n* = 422).

Variables	Mean	SD	S	K	Kolmogorov-Smirnov		Shapiro-Wilk	
					D	*P*	W	*P*
Social media use	3.50	0.689	0.175	0.597	0.031	0.673	0.994	0.306
Body image dissatisfaction	2.13	0.563	0.076	−0.063	0.025	0.927	0.997	0.847
Physical activity	1446.08	880.940	0.621	−0.094	0.069	0.002**	0.964	0.000**
Dietary behavior	1.35	0.705	−0.007	0.211	0.027	0.862	0.997	0.919

**P < 0.01.

To assess the clinical relevance of eating behavior problems in the sample, we categorized participants according to established clinical thresholds on the Dutch Eating Behavior Questionnaire (Table 3). The results showed that a significant proportion of participants exhibited clinically significant eating behavior problems. Specifically, 27.5% of participants (*n* = 116) scored above the threshold (>2.75) on the Emotional Eating subscale, indicating a tendency to use eating to cope with negative emotions. Furthermore, 33.2% of participants (*n* = 140) scored high on the External Eating subscale (>3.20), suggesting their eating behavior was easily driven by external food cues (such as seeing or smelling food). Finally, 18.7% of participants (*n* = 79) showed high levels (>3.25) on the Restrictive Eating subscale, reflecting their strong cognitive control over weight and body shape. These data suggest that this study sample includes a noteworthy subgroup at risk of eating pathology.

**TABLE 3:** Descriptive and clinical characteristics of dietary behavior and social media use (*n* = 422).

Characteristic	Value
**Dietary behavior (DEBQ) - clinical cut-offs**	
Participants above clinical cut-off for Emotional Eating (>2.75)	116 (27.5%)
Participants above clinical cut-off for External Eating (>3.20)	140 (33.2%)
Participants above clinical cut-off for Restrained Eating (>3.25)	79 (18.7%)
**Social media use**	
Average daily duration (hours), mean (SD)	2.8 (1.1)
Frequency of checking: multiple times per day	305 (72.3%)
**Frequent exposure to content types**	
Fitness, health, or diet-related content	276 (65.4%)
Food, restaurant, or “mukbang” content	245 (58.1%)

Characteristics of social media use descriptive data on social media use showed that participants spent an average of 2.8 ± 1.1 h per day on social media. Regarding the most frequently accessed content types (based on frequency statistics of relevant items in the eight social media questionnaires), 65.4% of participants reported frequently or very frequently browsing fitness, health, or diet-related content, while 58.1% frequently accessed food, restaurant, or “mukbang” (eating broadcast) content. Furthermore, 72.3% of participants indicated they checked social media multiple times a day. These data paint a picture of a young population highly immersed in the social media environment and frequently exposed to content directly related to body image and diet, providing important background information for the mediation mechanisms explored in this study.

### 3.2 Variables correlation analysis

**FIGURE 2:**
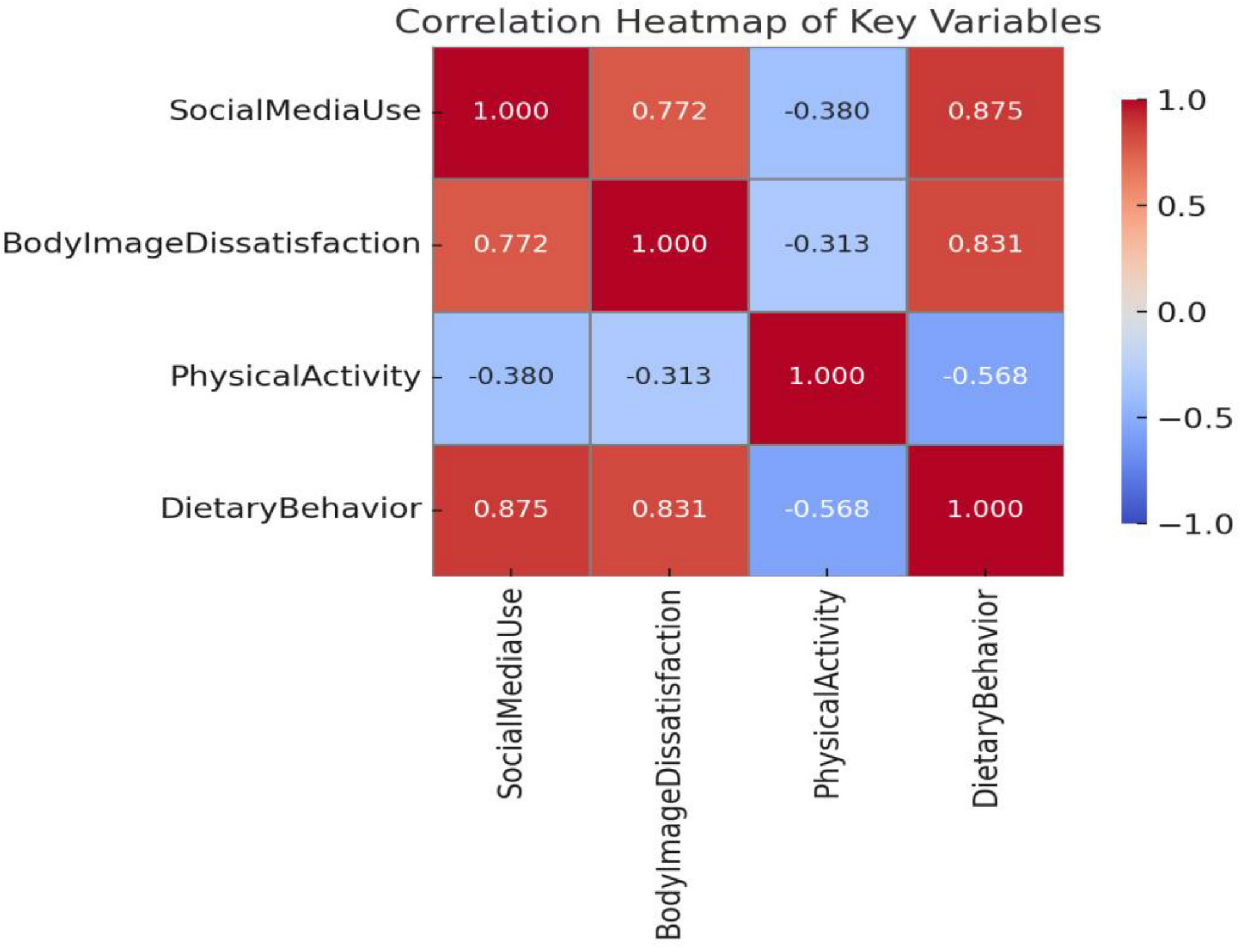
Heat map of correlations between variables. Red represents positive correlation, blue represents negative correlation. The darker the color, the higher the correlation coefficient.

Pearson correlation coefficients among the key study variables are presented in Table 4 and Figure 2. As expected, social media use was positively and strongly correlated with body image dissatisfaction (*r* = 0.707, *p* < 0.001), suggesting that higher engagement with social media is associated with greater concern over body appearance.

**TABLE 4:** Correlation analysis.

Variables	Social media use	Body image dissatisfaction	Physical activity	Dietary behavior
Social media use	1	–	–	–
Body image dissatisfaction	0.707**	1	–	–
Physical activity	−0.273**	−0.210**	1	–
Dietary behavior	0.584**	0.594**	−0.443**	1

**P < 0.001.

Social media use also demonstrated a moderate positive correlation with dietary behavior problems (*r* = 0.584, *p* < 0.001), indicating that individuals who reported higher levels of social media engagement were more likely to exhibit disordered eating patterns. Conversely, it was negatively associated with physical activity (*r* = −0.273, *p* < 0.001), suggesting a potential behavioral displacement or demotivating effect.

In parallel, body image dissatisfaction was negatively correlated with physical activity (*r* = −0.210, *p* < 0.001), and positively correlated with problematic dietary behavior (*r* = 0.594, *p* < 0.001), further supporting its role as a psychological risk factor. Finally, physical activity was negatively associated with dietary behavior issues (*r* = −0.443, *p* < 0.001), suggesting a potential protective effect of regular exercise against unhealthy eating.

These results provide initial support for the hypothesized relationships among variables and validate the rationale for conducting the proposed parallel mediation analysis.

### 3.3 Parallel mediation model analysis

Confirmatory factor analysis (CFA) was conducted to evaluate the validity of the measurement model. As shown in Table 5, all observed indicators loaded significantly onto their respective latent variables, with standardized factor loadings ranging from 0.69 to 0.84 (all *p* < 0.001), indicating satisfactory convergent validity.

**TABLE 5:** Factor loadings of all indicators.

Variables	Item content	Standardized loading (λ)
SMU1	How many hours per day do you spend on social media?	0.81
SMU2	How often do you check social media before sleeping?	0.79
SMU3	How often do you view fitness- or diet-related posts?	0.78
SMU4	How often do you compare your body with others online?	0.76
SMU5	How often do you follow health influencers?	0.74
SMU6	How often do you engage with body-related content?	0.72
SMU7	How frequently do you seek diet advice online?	0.7
SMU8	How often do you feel influenced by social media images?	0.69
BSQ1	Have you felt ashamed of your body?	0.84
BSQ2	Have you avoided looking at yourself in the mirror?	0.83
BSQ3	Have you felt excessively concerned about your weight?	0.82
BSQ4	Have you compared your body negatively with others?	0.8
BSQ5	Have you avoided social events because of your appearance?	0.79
BSQ6	Have you worn baggy clothes to hide your body?	0.78
BSQ7	Have you worried about others judging your shape?	0.77
BSQ8	Have you felt distressed shopping for clothes?	0.76
BSQ9	Have you been afraid to eat for fear of gaining weight?	0.75
BSQ10	Have you wished to be thinner to feel accepted?	0.74
BSQ11	Have you avoided eating in public due to body concerns?	0.73
BSQ12	Have you weighed yourself obsessively?	0.72
BSQ13	Have you experienced anxiety about body image?	0.71
BSQ14	Have you disliked photos of yourself?	0.7
BSQ15	Have you tried to conceal specific body parts?	0.69
BSQ16	Have you engaged in body-checking behaviors?	0.68
DEBQ	Emotional eating	0.8
	External eating	0.795
	Restrained eating	0.79

Collectively, these fit indices provide strong support for the construct validity and factorial structure of the latent variables included in the study (i.e., physical activity, sleep quality, smartphone addiction, and cognitive failures). The results indicate that the measurement model adequately represents the observed data and is suitable for further structural equation modeling.

Model fit indices (Table 6) suggested that the four-factor measurement model demonstrated acceptable to good model fit: χ^2^/df = 1.92, CFI = 0.926, TLI = 0.912, RMSEA = 0.057, and SRMR = 0.048. All indices met or exceeded commonly accepted thresholds, supporting the structural adequacy of the hypothesized model. The results indicate that the measurement model adequately represents the observed data and is suitable for further structural equation modeling.

**TABLE 6:** Model fit indices for the measurement model.

Fit index	Value	Acceptable threshold
χ^2^/df	1.92	<3.00
Comparative fit index (CFI)	0.926	≥0.90
Tucker–Lewis index (TLI)	0.912	≥0.90
Root mean square error of approximation (RMSEA)	0.057	≤0.08
Standardized root mean square residual (SRMR)	0.048	≤0.08

**FIGURE 3:**
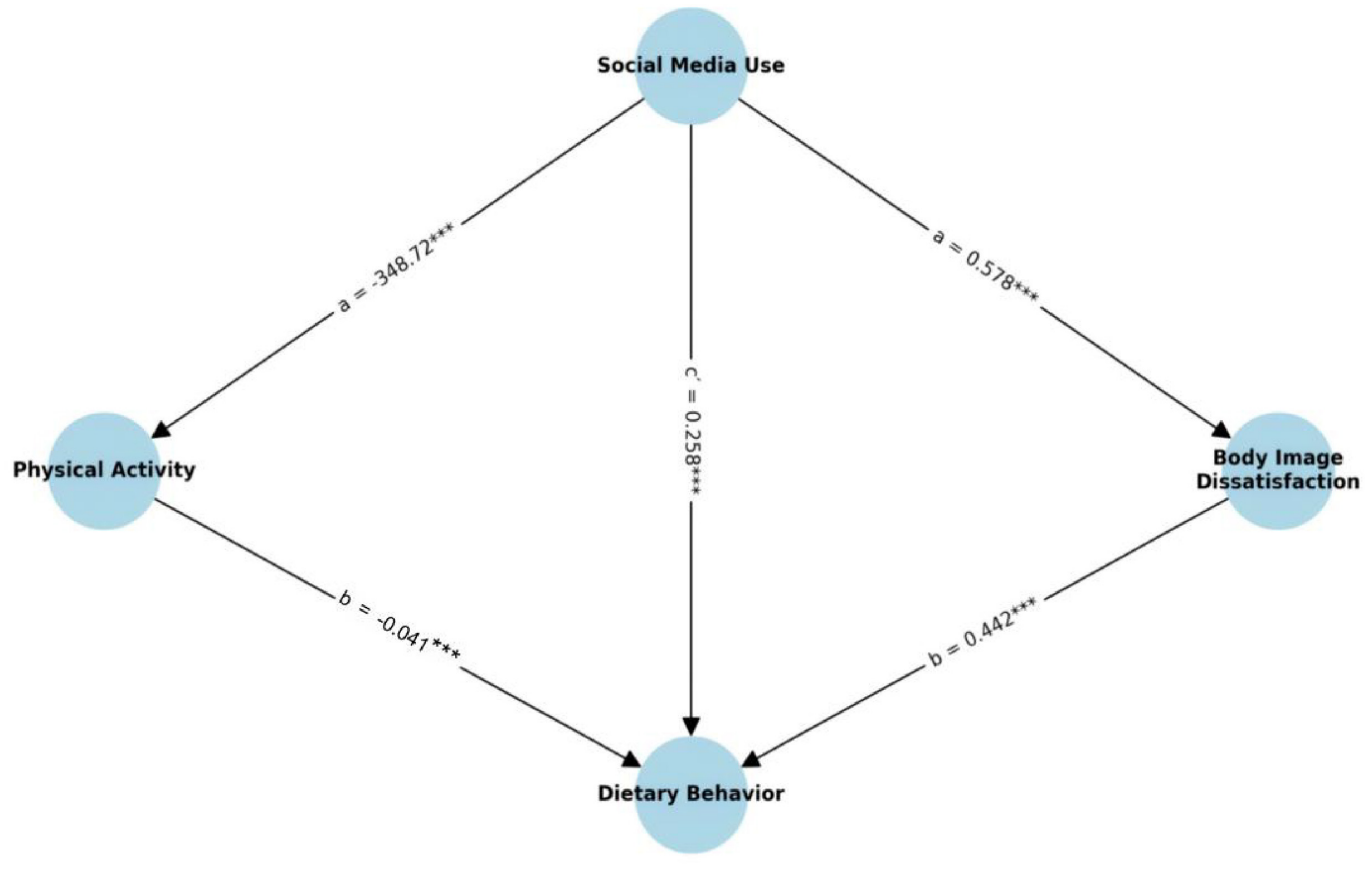
Parallel mediation model path diagram. Values represent the significant standardized regression coefficients. ***p < 0.01.

A parallel mediation model was conducted to test whether the relationship between social media use and dietary behavior was mediated by body image dissatisfaction and physical activity, respectively. The results are presented in Figure 3 and Table 7.

**TABLE 7:** Bootstrap analysis of the mediation effect size and significance test.

Path			Standardized effect size (effect)	95% CI		*P*
				LL	UL	
Social media use ≥ body image dissatisfaction ≥ dietary behavior	a*b	Indirect effect	0.256**	0.169	0.335	0.000
Social media use ≥ body image Dissatisfaction	a	X ≥ M	0.578**	0.512	0.644	0.000
Body image Dissatisfaction = > Dietary Behavior	b	M ≥ Y	0.442**	0.298	0.586	0.000
Social media use ≥ dietary behavior	c’	Direct effect	0.258**	0.138	0.378	0.000
Social media use ≥ dietary behavior	c	Total effect	0.597**	0.503	0.692	0.000
Social media use ≥ physical Activity = > Dietary Behavior	a*b	Indirect effect	0.084**	0.043	0.125	0.000
Social media use ≥ physical activity	a	X ≥ M	−348.717**	–488.395	–	0.000
Physical activity ≥ dietary behavior	b	M ≥ Y	−0.041**	−0.007	−0.089	0.000
Social media use ≥ dietary behavior	c’	Direct effect	0.258**	0.138	0.378	0.000
Social media use ≥ dietary behavior	c	Total effect	0.597**	0.503	0.692	0.000

**P < 0.01. CI confidence interval; LL, lower limit; UL, upper limit. X, independent variable; M, mediating variable; Y, dependent variable. Bolded values represent significant differences.

The indirect effect of social media use on dietary behavior through body image dissatisfaction was statistically significant (ab = 0.256, 95% CI [0.169, 0.335], SE = 0.043, *z* = 5.965, *p* < 0.001). Specifically, social media use significantly predicted body image dissatisfaction (*a* = 0.578, 95% CI [0.512, 0.644], *p* < 0.001), and body image dissatisfaction, in turn, significantly predicted dietary behavior (*b* = 0.442, 95% CI [0.298, 0.586], *p* < 0.001). This suggests a strong psychological mediation pathway through increased body dissatisfaction.

The mediation through physical activity was also statistically significant but smaller in magnitude (ab = 0.084, 95% CI [0.043, 0.125], SE = 0.021, *z* = 4.059, *p* < 0.001). Social media use negatively predicted physical activity (*a* = −348.717, 95% CI [−488.395, −209.040], *p* < 0.001), and physical activity negatively predicted dietary behavior (*b* = −0.041, CI [−0.007, −0.089], *p* < 0.001), indicating that reduced physical activity serves as a behavioral risk pathway from media exposure to eating issues.

Even after accounting for both mediators, the direct effect of social media use on dietary behavior remained significant (c’ = 0.258, 95% CI [0.138, 0.378], *p* < 0.001), suggesting partial mediation. The total effect of social media use on dietary behavior was c = 0.597 (95% CI [0.503, 0.692], *p* < 0.001).

**FIGURE 4:**
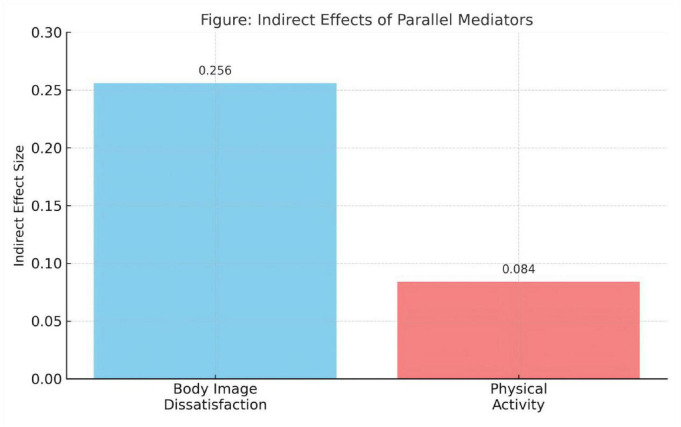
Comparison of indirect effect values of mediating variables.

These findings confirm that both body image dissatisfaction and physical activity independently mediate the impact of social media use on dietary behavior, supporting the hypothesized parallel mediation model. Notably, the psychological pathway (via body image dissatisfaction) contributed a larger proportion of the total indirect effect compared to the behavioral pathway (via physical activity) (See Figure 4).

To verify whether the parallel mediation model (Model A) proposed in this study is the optimal explanatory model, we compared it with a series of theoretically meaningful alternative models. These alternative models included: (1) a direct effects model: a model without any mediating variables (social media use → eating behavior); (2) a body image dissatisfaction-only mediation model; and (3) a sequential mediation model: social media use → body image dissatisfaction → physical activity → eating behavior. We calculated the Akaike Information Criterion (AIC) and Bayesian Information Criterion (BIC) values for each model using structural equation modeling. Lower AIC and BIC values indicate a better balance between complexity and fit. As shown in Table 8, the parallel mediation model (Model A) in this study has the lowest AIC and BIC values, and other fit indices (CFI, TLI, RMSEA) also perform well, indicating that it is the optimal and simplest model for the relationship between explanatory variables.

**TABLE 8:** Fit indices for alternative model comparisons.

Model Description	χ ^2^/df	CFI	TLI	RMSEA	SRMR	AIC	BIC
Model A: proposed parallel mediation	1.92	0.926	0.912	0.057	0.048	4123.75	4281.34
Model B: direct effect model (no mediation)	2.85	0.872	0.855	0.081	0.067	4189.42	4301.56
Model C: body image dissatisfaction-only mediation	2.15	0.908	0.894	0.065	0.055	4145.18	4290.11
Model D: serial mediation model	2.08	0.915	0.901	0.062	0.058	4138.92	4295.81

### 3.4 ANOVA of variables in different physical activity groups

To examine whether levels of physical activity were associated with differences in key variables, one-way ANOVAs were conducted comparing Social Media Use, Body Image Dissatisfaction, and Dietary Behavior across three physical activity levels: Low, Moderate, and High, based on IPAQ classification (See Table 9).

**TABLE 9:** Analysis of variance for different physical activity level groups.

Variables	PA_level (mean ± SD)			F	*P*
	High (*n* = 81)	Low (*n* = 95)	Moderate (*n* = 246)		
Social media use	3.04 ± 0.80	3.82 ± 0.62	3.46 ± 0.66	11.326	0.000**
Body image dissatisfaction	1.76 ± 0.59	2.36 ± 0.57	2.11 ± 0.54	9.329	0.000**
Dietary behavior	0.37 ± 0.71	1.72 ± 0.68	1.34 ± 0.63	31.460	0.000**

**P < 0.01.

There was a statistically significant difference in social media use across activity levels [F(2, 297) = 11.326, *p* < 0.001]. Participants in the low physical activity group reported the highest level of social media use (*M* = 3.82, SD = 0.62), significantly higher than both the moderate group (*M* = 3.46, SD = 0.66) and the high activity group (*M* = 3.04, SD = 0.80). This suggests an inverse relationship between physical activity and social media engagement.

Similarly, body image dissatisfaction significantly differed by physical activity level [F(2, 297) = 9.329, *p* < 0.001). Individuals in the low activity group exhibited the highest levels of dissatisfaction (*M* = 2.36, SD = 0.57), followed by those in the moderate (*M* = 2.11, SD = 0.54) and high (*M* = 1.76, SD = 0.59) groups. These findings support the notion that higher levels of physical activity may serve as a protective factor against negative body image perceptions.

The most pronounced group difference was observed in dietary behavior [F(2, 297) = 31.460, *p* < 0.001]. Participants with low physical activity reported the most problematic eating patterns (*M* = 1.72, SD = 0.68), compared to those with moderate (*M* = 1.34, SD = 0.63) and high (*M* = 0.37, SD = 0.71) levels of activity. These results highlight a strong behavioral gradient, indicating that physical activity may play a substantial role in mitigating disordered eating.

**FIGURE 5:**
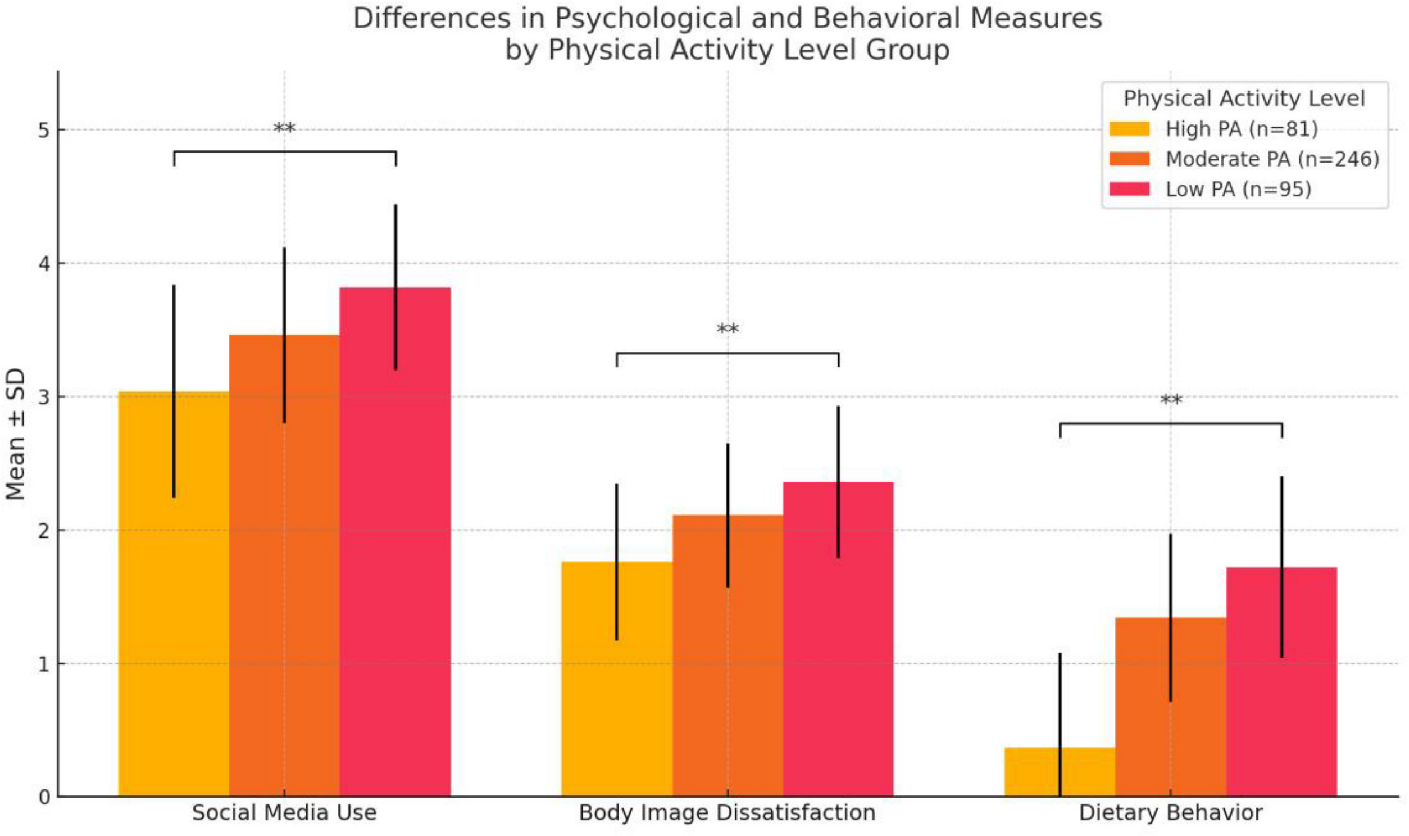
ANOVA bar chart of different physical activity groups. ** Indicates significant difference.

Figure 5 illustrate the distribution of Social Media Use, Body Image Dissatisfaction, and Dietary Behavior scores across three physical activity levels. Significant group differences were observed for all three variables, with more disordered profiles concentrated in the Low physical activity group.

## 4 Discussion

This study investigated the relationship between social media use and disordered dietary behavior among Chinese young adults, focusing on the parallel mediating roles of body image dissatisfaction and physical activity. Utilizing a structural equation modeling approach, we found that social media use significantly predicted both disordered eating behavior and the two proposed mediators. Specifically, body image dissatisfaction emerged as a strong positive mediator, while physical activity served as a modest but significant negative mediator. These findings confirm the hypothesized parallel mediation model and provide new insights into how social media affects dietary behavior through co-occurring psychological and behavioral pathways.

### 4.1 The dual impact of social media on dietary behavior

Consistent with previous literature, social media use was found to be significantly and positively associated with problematic dietary behavior. Individuals who reported greater engagement with social media particularly appearance- and food-related content exhibited higher tendencies toward emotional, external, and restrained eating (Wu et al., 2024; Barklamb et al., 2020). This supports the idea that frequent exposure to idealized images and food-related stimuli on social platforms is linked to disruptions in normative eating patterns, as well as higher levels of body-related anxiety and food salience and cravings.

Our findings extend existing knowledge by demonstrating that this relationship is not merely direct but operates through two distinct and concurrent mediating mechanisms: body image dissatisfaction and physical activity engagement. The psychological pathway, represented by body image dissatisfaction, emerged as the stronger mediator, underscoring the centrality of self-perceptual processes in the etiology of disordered eating. Individuals who perceived greater discrepancies between their own bodies and those portrayed on social media were more likely to engage in maladaptive eating practices, a finding that aligns with Social Comparison Theory and a wealth of empirical research on media-induced body dissatisfaction (So and Kwon, 2023). Our findings are corroborated by a recent systematic review which concluded that exposure to body- and food-related content on social media is robustly linked to stronger eating desires and more disordered eating behaviors, underscoring the pervasive nature of these digital influences (Wu et al., 2024).

### 4.2 Dual pathways from social media to disordered eating

This dual-pathway approach offers a more comprehensive understanding of the link between digital media exposure and health-related behavior. While previous studies have often focused on a single mediation route, typically emphasizing psychological outcomes such as body image or self esteem (Palmeira et al., 2009; Friedman et al., 2002). Our findings highlight the concurrent roles of body image dissatisfaction and physical activity engagement, each contributing independent explanatory power. For instance, a biopsychosocial study on young adults highlighted that diet and mental health are intertwined through complex pathways involving both psychological state and health behaviors (Rossa-Roccor et al., 2021). Our model operationalizes this complexity by simultaneously modeling cognitive-affective and behavioral mediators, providing a more holistic framework than earlier, simpler models.

The results revealed that body image dissatisfaction accounted for the majority of the total indirect effect, emphasizing its critical role as a psychological conduit. At the same time, the behavioral pathway through reduced physical activity, although smaller in effect size remained significant, supporting a multifactorial view of disordered eating wherein both internal perceptions and external behaviors are shaped by media environments. This distinction is important for health interventions: addressing only psychological factors (e.g., self esteem, body image) without considering behavioral displacement (e.g., sedentary lifestyles) may yield suboptimal results. Our findings support a biopsychosocial framework, where cognitive emotional factors and lifestyle practices interact in complex but measurable ways (Rossa-Roccor et al., 2021).

### 4.3 Psychological mechanisms: social comparison and body image dissatisfaction

The strong mediating effect of body image dissatisfaction reinforces the principles of Social Comparison Theory (Festinger, 1957), which posits that individuals evaluate themselves in relation to others, especially in environments lacking objective standards. On image centric platforms such as Instagram or TikTok, young people are exposed to curated, filtered, and often idealized portrayals of thinness, fitness, and dietary “success,” which serve as potent social mirrors. These upward comparisons may distort self-perception and heighten dissatisfaction with one’s own appearance, thereby driving unhealthy eating behaviors such as restrictive dieting, binge eating, or emotional overeating (Zaharia and Gonţa, 2024; Charinsarn and Nuttavuthisit, 2025).

Importantly, our study operationalizes these theoretical concepts within a mediation structure. Rather than treating body dissatisfaction as a static outcome, we model it as an active transmission mechanism through which social media indirectly affects dietary behavior. This distinction offers practical value: psychological interventions targeting body image may have downstream benefits for dietary regulation.

### 4.4 Behavioral mechanisms: the role of physical activity

The indirect pathway via physical activity aligns with Self-Determination Theory, which emphasizes the importance of intrinsic motivation, autonomy, and competence in sustaining health related behaviors (Deci and Ryan, 2012). While social media has the potential to inspire physical activity through exposure to fitness routines or motivational content, its effects are conditional. For some users, especially those with lower intrinsic motivation or poorer self-image, exposure to “fitspiration” may lead to alienation, inadequacy, or demotivation (Jerónimo and Carraça, 2022).

The negative association between social media use and physical activity observed in our study may reflect two overlapping phenomena. First, consistent with the displacement hypothesis, increased screen time may reduce opportunities for active behavior (Twenge et al., 2018). Second, the demoralizing effects of unattainable body ideals may undermine motivation to engage in physical activity altogether. Both pathways highlight the non-neutral nature of digital exposure. It is embedded in motivational dynamics that influence real-world health practices.

Moreover, our parallel model contrasts with prior research that often assumes a serial mediation structure (e.g., social media → body image → physical activity → eating) (Shi et al., 2024). Instead, we demonstrate that body image dissatisfaction and physical activity operate as distinct but simultaneous mediators, a perspective with significant implications for designing multi-component interventions.

### 4.5 Physical activity as a moderator: group-level insights

Beyond mediation, our study also examined how physical activity level moderates key variables. Participants with low physical activity reported significantly higher levels of social media use, body dissatisfaction, and disordered eating compared to their moderate and high activity peers. These group differences provide further evidence for the protective role of physical activity—not just as a behavioral outcome but as a psychosocial buffer. Individuals in the high activity group showed the lowest levels of disordered eating and body dissatisfaction, suggesting that consistent physical activity may foster positive body-related self-perceptions and better appetite regulation. These results align with previous findings that regular exercise enhances self-efficacy, reduces depressive symptoms, and improves self regulation of eating (Borden and Cook-Cottone, 2020). Interestingly, the highest levels of social media use were observed in the low physical activity group, supporting the displacement hypothesis and potentially reflecting psychological profiles predisposed to both sedentary behavior and digital over-engagement. Such findings reinforce the need for intervention strategies that jointly address both screen time and active lifestyle promotion (Mutz et al., 1993).

### 4.6 Theoretical and practical implications

These findings offer several contributions to theoretical models and public health practices. Theoretically, the parallel mediation model enriches traditional unidimensional frameworks by integrating both internal (body image) and external (behavioral engagement) factors. This model is consistent with biopsychosocial approaches (Sarafino and Smith, 2014), which recognize the interplay of psychological, behavioral, and environmental influences on health behavior. Practically, digital health literacy programs should train youth to critically interpret online content, recognizing curated and potentially harmful portrayals of body image and dietary practices. Psychological interventions, such as cognitive-behavioral therapy, targeting body dissatisfaction could yield secondary benefits for eating regulation (Linardon et al., 2017). Fitness-focused media campaigns should promote body diversity, intrinsic motivation, and realistic goals, avoiding guilt-inducing or appearance-centric messages. Policy level actions could include content regulation or disclaimers for posts that promote unscientific or potentially harmful dietary practices, similar to existing regulations on alcohol, tobacco, or cosmetic advertising.

### 4.7 Limitations

Despite the valuable contributions of this study, several limitations warrant consideration. The cross-sectional design prevents the establishment of causal relationships; thus, longitudinal or experimental studies are essential to determine temporal precedence and potential feedback loops (e.g., whether changes in dietary behavior influence subsequent social media usage). The reliance on self-report measures introduces potential biases such as recall bias and social desirability, which could be mitigated by incorporating objective metrics (e.g., wearable devices for physical activity, food diaries). Additionally, the effects of specific social media platforms (e.g., Instagram, TikTok, YouTube) may differ and warrant disaggregation in future studies. Incorporating moderating variables such as gender identity, body mass index, and media literacy could provide deeper insights into the conditions under which social media exerts the greatest impact.

## 5 Conclusion

This study provides empirical support for a parallel mediation model connecting social media use to disordered dietary behavior in young adults through two distinct yet concurrent pathways: body image dissatisfaction and physical activity engagement. The findings underscore the psychological impact of appearance based social comparisons and the behavioral consequences of sedentary media consumption. While body image dissatisfaction emerged as the stronger mediator, reduced physical activity also significantly contributed to unhealthy eating patterns. By integrating both psychological and behavioral mechanisms, this study offers a more nuanced understanding of how digital environments shape health-related behavior. The results highlight the need for multifaceted interventions that simultaneously promote digital media literacy, positive body image, and active lifestyles. Future research should adopt longitudinal designs and explore additional moderators to further clarify these relationships across diverse populations.

## Data Availability

The original contributions presented in this study are included in this article/Supplementary material, further inquiries can be directed to the corresponding authors.
